# Biomechanical impact of cortical bone vs. traditional pedicle screw trajectories: a finite element study on lumbar spinal instrumentation

**DOI:** 10.3389/fbioe.2025.1541114

**Published:** 2025-02-18

**Authors:** Xishan Li, Khaled H. A. Abdel-Latif, Jefrem Schwab, Xiang Zhou, Jie Yang, Zully M. Ritter, Arndt F. Schilling, Maximilian Reinhold

**Affiliations:** ^1^ Department of Trauma Surgery, Orthopedics and Plastic Surgery, University Medical Center Göttingen, Göttingen, Germany; ^2^ Department of Otorhinolaryngology, Head and Neck Surgery, Faculty of Medicine and University Hospital Cologne and Jean Uhrmacher Institute for Clinical ENT-Research, University of Cologne, Köln, Germany; ^3^ Deparment of Medical Informatics, University Medical Center Göttingen, Göttingen, Germany

**Keywords:** traditional pedicle screw trajectory, cortical bone trajectory, finite element analysis, lumbar spine instrumentation, screw breakage

## Abstract

**Background:**

Pedicle screw fixation using the cortical bone trajectory (CBT) enhances stability by engaging cortical bone, offering a valuable alternative to the traditional pedicle screw trajectory (TT). This study used finite element analysis to compare L4-5 instrumentation with CBT and TT screws, investigating whether the increased cortical bone engagement in CBT improves stability but makes it more susceptible to fatigue failure.

**Methods:**

A L3-sacrum model was generated using anonymized CT patient data, validated against existing studies, showing consistent ROM (range of motion) values. A mono-segmental L4-5 instrumentation with an interbody fusion cage was configured with both TT and CBT models, differentiated for healthy and osteoporotic bone (reduced Young’s modulus). Both models were exposed to simulated biomechanical loading conditions (compression, flexion, extension, lateral bending, and rotation) to calculate screw loosening and breakage risk. Screw loosening was assessed by measuring micro-movements within the screw hole, while screw breakage was evaluated based on maximum stress values and their frequency at the same locations.

**Results:**

In both healthy and osteoporotic bone, the CBT model exhibited smaller micro-movements compared to the TT model across all motions. For maximum stress in healthy bone, CBT showed lower stress during right rotation but higher stress in the other six motions. In osteoporotic bone, CBT stress exceeded TT stress in all conditions. The TT model in healthy bone showed stress concentrations at three locations, while CBT distributed stress across five sites. In osteoporotic bone, CBT showed stress at three locations, while TT distributed stress at four. Notably, in the TT model, maximum stress occurred at the screw head in six of seven movements, whereas in the CBT model, three movements showed maximum stress at the screw head and three at the screw tail.

**Conclusion:**

CBT screws, by traversing three cortical layers, achieve greater integration with the vertebral bone compared to TT screws, thus reducing the risk of screw loosening. Although this increases the maximum stress on the screws, the stress is more evenly distributed, with the screw tail helping to reduce the risk of breakage.

## 1 Introduction

Pedicle screw fixation is the most common technique for stabilizing the lumbar spine, providing strong support for correcting deformities, stabilizing fractures, treating tumors, and managing other spinal pathologies, with the aim of promoting durable lumbar interbody fusion (Bydon et al., 2014; Chen et al., 2020). Advancements in internal fixation techniques have led to the development of various intervertebral fusion methods over time. One recent advancement is the cortical bone trajectory (CBT), an alternative to the traditional trajectory (TT). The CBT technique is characterized by following a medial-to-lateral and upward screw trajectory, that increases screw contact with denser cortical bone, thus enhancing construct stability especially in osteoporotic patients. In contrast, the TT approach relies on a lateral-to-medial trajectory through the pedicle and vertebral body, primarily engaging cancellous bone, which result in reduced implant stability in cases of compromised bone quality according to the literature (Santoni et al., 2009; Matsukawa et al., 2013; Baluch et al., 2014; Kim et al., 2022).

Nevertheless, it remains unclear whether CBT provides superior biomechanical properties such as pullout and fixation strength compared to TT methods (Santoni et al., 2009; Matsukawa et al., 2016; Zhang et al., 2019). There is inconsistency in the reported results that may stem from the widespread use of vertical pullout tests, which do not accurately reflect real-life spinal movements. Additionally, there has been limited research on the fatigue performance of different insertion techniques, with existing studies failing to show any clear advantage for CBT (Akpolat et al., 2016). This raises a critical concern: the increased cortical bone contact in CBT, which theoretically enhances stability, may also make it more prone to fatigue failure.

In this study, a finite element analysis (FEA) model was developed to simulate realistic conditions for a common mono-segmental L4-5 lumbar spinal instrumentation, with the use of either CBT or TT pedicle screw fixation techniques, alongside a lumbar interbody fusion device (cage) in both groups. We primarily evaluated screw micro-movements, the internal fixation device’s maximum stress values, and the locations of maximum stress occurrences. These metrics provided an assessment and comparison of construct stability, screw loosening risk, and implant failure likelihood—such as screw breakage—to improve understanding of CBT techniques and their clinical application.

## 2 Materials and methods

### 2.1 Materials

#### 2.1.1 CT data

A 31-year-old male patient underwent radiological evaluation at the Department of Radiology, University Medical Centre Göttingen, which revealed no tumor, deformity or fracture in the lumbosacral spine.

#### 2.1.2 Workstation specifications

The computational setup included an Intel(R) Core(TM) i9-10900K CPU @ 3.70 GHz, 64 GB of RAM, and an Nvidia Quadro RTX 4000 graphics card (8.0 GB).

#### 2.1.3 Software

The following software tools were utilized: 3D Slicer 5.6.2 (https://www.slicer.org), Geomagic Wrap 2021 (3D Systems, United States), SolidWorks 2018 (Dassault Systèmes, France), and ANSYS 2021R2 (ANSYS, United States).

### 2.2 Methods

#### 2.2.1 Model development and groups

##### 2.2.1.1 3D reconstruction of the L3-sacrum

CT data was first imported into 3D Slicer software. The ‘Volume Rendering’ module was accessed with the ‘CT-bone’ preset, and the ‘shift’ was adjusted to display the complete 3D bone model. Each vertebra and the sacrum were manually adjusted, and the volume was cropped using the “Crop Volume” model. Different colors were then applied to distinguish the various bones. Using the “Grow from Seeds” function, the marked structures were filled and separated, and the 3D surface models of each bone were exported and checked in Geomagic Wrap.

The bone model, derived from 3D Slicer, was imported into Geomagic Wrap, revealing an exterior composed of triangular facets and an interior cluttered with disorganized triangular faces. The first step was to refine the exterior surface and remove the interior clutter, which was accomplished using the ‘Polygons’ module. Next, the ‘Exact Surfaces’ module was accessed, where ‘Contours’, ‘Patches,’ and ‘Grids’ were used to refine and optimize the mesh iteratively. Finally, ‘Fit Surfaces’ was applied to generate the 3D solid model of each bone. The cortical and trabecular bone models were then created for each bone using the ‘Offset Entire Model’ function.

Since all vertebrae originated from the same CT scan, they shared a common coordinate origin. Each bone model was imported into SolidWorks, and the assembly of the lumbosacral model was completed by aligning the models using this common origin. Based on this assembled model, facet cartilage and intervertebral discs (comprising the cartilaginous endplate, annulus fibrosus, and nucleus pulposus) were constructed in SolidWorks through sketch-based modeling. This process resulted in a detailed spinal model spanning from the L3 vertebra to the sacrum, accurately reflecting the spine’s anatomical structure and functional dynamics.

##### 2.2.1.2 3D reconstruction of pedicle screw and rod fixation system

Using the sketch-based modeling method in SolidWorks, the pedicle screw and rod fixation system was constructed, including the screw, connecting rod, and interbody fusion cage. These devices were reviewed by Dr. Reinhold to ensure compliance with surgical requirements and a precise fit with the CT model. The specifications included screws with a length of 55 mm, with the L4 screw having a width of 5.5 mm and the L5 screw being slightly narrower at 4.5 mm. The connecting rod had a width of 5.5 mm, and the interbody fusion cage measured 26 mm × 10 mm × 10 mm.

##### 2.2.1.3 Traditional trajectory (TT) model

**FIGURE 1:**
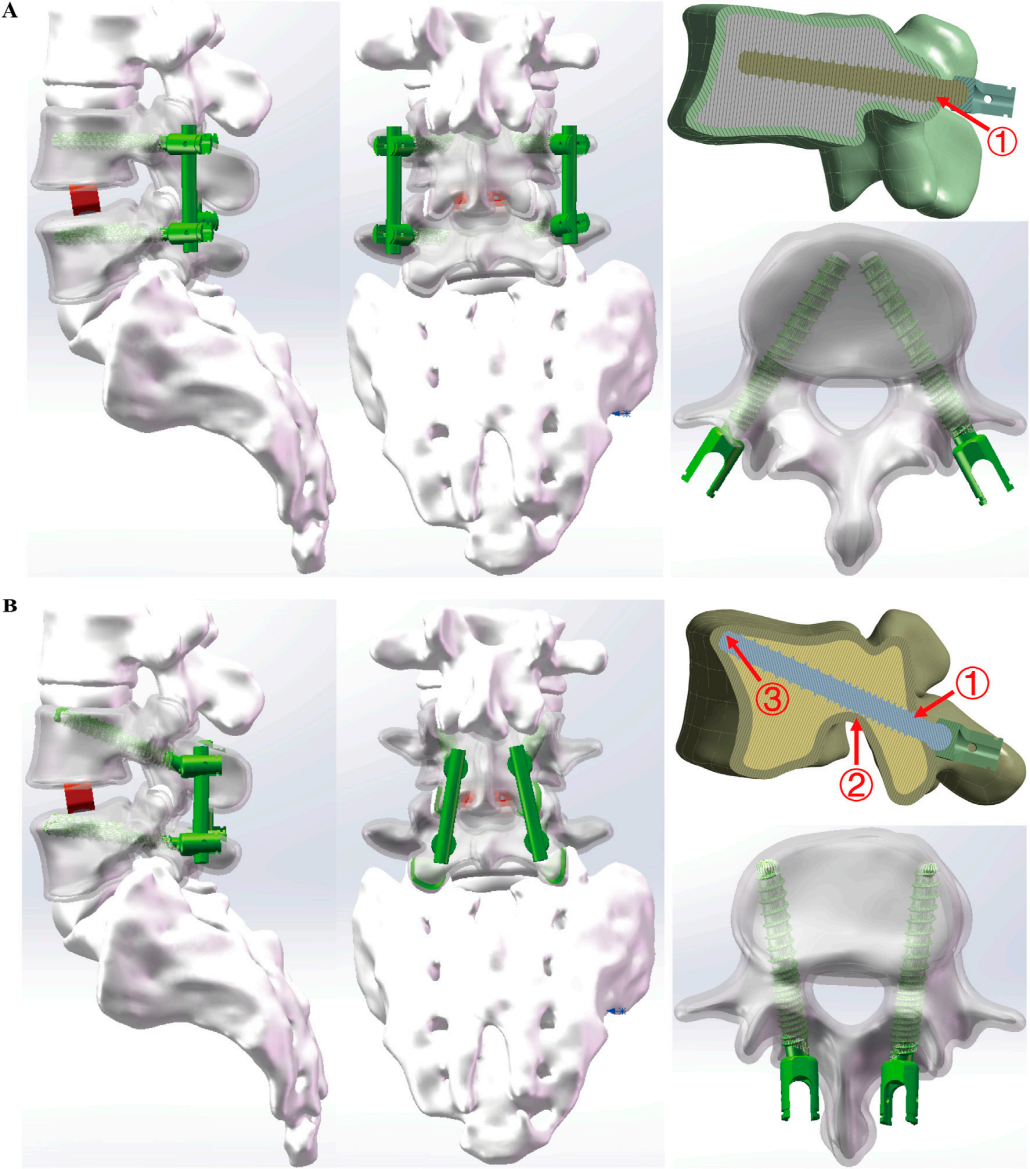
Two Strategies for Stabilizing Lumbar Adjacent Segment Disease. **(A)** TT (traditional trajectory) model in different views, contacting one layer of cortical bone. **(B)** CBT (cortical bone trajectory) model in different views, contacting three layers of cortical bone. Both models utilized screws with a width of 5.5 mm at the L4 vertebra and 4.5 mm at the L5 vertebra. The length of all screws was 55 mm. The interbody fusion cage dimensions were identical in both models, measuring 26 mm × 10 mm × 10 mm.

To simulate posterior lumbar interbody fusion, the intervertebral disc at the L4/5 level was removed and replaced with a rigid fusion cage. In the TT model, screws were implanted at the intersection of the horizontal line from the midpoint of the transverse process and the vertical edge of the superior articular process, as described by Magerl and Dick (Dick, 1984; Dick et al., 1985). The screws followed the axis of the pedicle in the vertebral arch, entering medially from an external position in the sagittal plane (Figure 1A).

##### 2.2.1.4 Cortical bone trajectory (CBT) model

Conversely, in the CBT model, screws were positioned following an ideal cortical bone trajectory as described by Matsukawa et al. (Matsukawa et al., 2015a; Jarvers et al., 2021). The entry point was located approximately 1 mm below the inferior margin of the transverse process at the midline of the superior articular process. Screw placement extended from the 5 o’ clock to the 11–12 o’clock position on the left pedicle, and from the 7 o’clock to the 12–1 o’clock position on the right (Figure 1B).

#### 2.2.2 Material properties

In this study, the healthy and the osteoporosis bone, as well as the screw, rod, and cage, were modelled as homogeneous, isotropic materials with linear elastic properties, consistent with established research (Wu et al., 2022; Zhang et al., 2022; Fan et al., 2023; Kahaer et al., 2023; Zhang et al., 2023). The osteoporosis bone model was developed using the same geometry, with reduced bone quality achieved by decreasing the Elastic Modulus of Young (Che et al., 2022). The major ligament groups, including the anterior longitudinal ligament (ALL), posterior longitudinal ligament (PLL), ligamentum flavum (LF), supraspinous ligament (SSL), and interspinous ligament (ISL), were also incorporated. The material properties employed in this study were selected based on their relevance to the research objectives and their common application in the field. Specific material properties were derived from previous studies (Waters and Morris, 1973; Reilly and Burstein, 1975; Wu and Yao, 1976; Marchand and Ahmed, 1990; Goel et al., 1993; Lu et al., 1996; Fan et al., 2019; Che et al., 2022) and are listed in Table 1.

**TABLE 1:** The material properties.

Material	Young’s modulus (MPa)	Poisson’s ratio	Stiffness coefficient (N/mm)
Healthy cortical bone	12,000	0.3	
Healthy cancellous bone	100	0.2	
Osteoporotic cortical bone	8,040	0.3	
Osteoporotic cancellous bone	34	0.2	
Facet cartilage	24	0.4	
Cartilaginous endplate	23.8	0.4	
Annulus fibrosis	4.2	0.45	
Nucleus pulposus	1	0.49	
Cage	3,600	0.25	
Screw and Rod (Titanium)	110,000	0.3	
Anterior longitudinal ligament			15
Posterior longitudinal ligament			21
Ligament flava			20
Supraspinous ligament			18
Interspinous ligament			16

#### 2.2.3 Boundary and loading conditions

##### 2.2.3.1 Contact conditions

In our model, the cortical and cancellous bones were bonded, as were the screws to the cortical bone. The cage was bonded to the adjacent upper and lower vertebral bodies, with the intervertebral discs similarly secured. Small inter-articular contacts were modelled with a friction coefficient of 0.1, while a higher coefficient of 0.3 was applied between the cancellous bone and screws to more accurately simulate mechanical interactions and stress distributions (Panteli et al., 2015; Pei et al., 2023).

##### 2.2.3.2 Loading conditions

**FIGURE 2:**
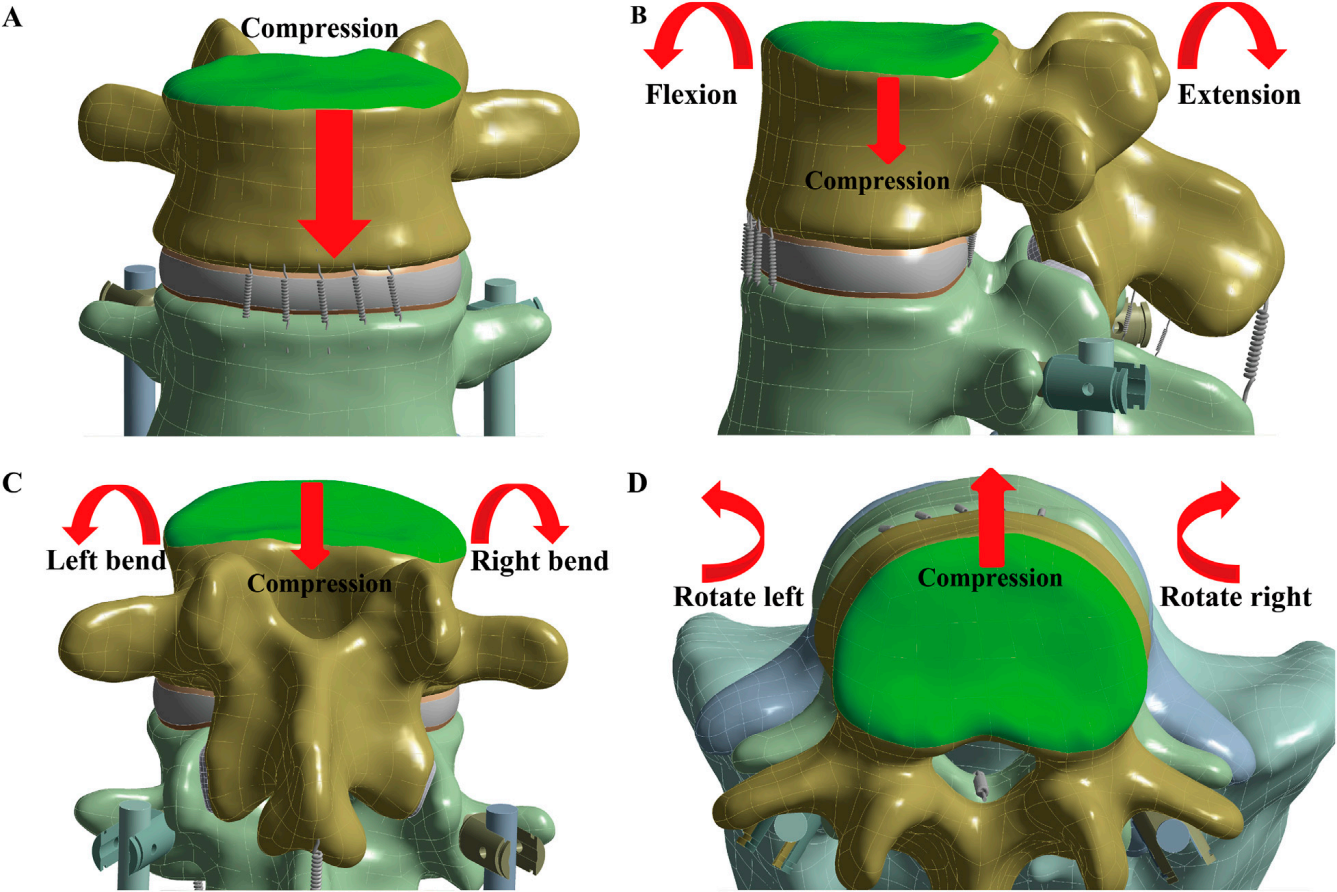
Simulate the seven lumbar movements. The loading position is at the L3 upper endplate (green), with the red arrow indicating the direction of the applied force. To simulate the seven lumbar movements, **(A)** Compression, **(B)** Flexion and Extension, **(C)** Left and Right bend, and **(D)** Left and Right rotation were applied.

During all simulations, the sacrum was fixed by constraining all degrees of freedom to prevent rigid body motion (Figure 2). A 900 N force, representing body weight, was applied to the upper endplate of L3 to model pure compression. For the six lumbar movements—flexion, extension, left and right lateral bending, and left and right rotation—a compressive load of 400 N and a torque of 7.5 Nm were applied to the upper endplate of L3 (Huang et al., 2023).

#### 2.2.4 Observed data

This study focuses on evaluating the risks of screw loosening and mechanical screw breakage. The risk of screw loosening was assessed by measuring micro-movements, quantified as the sliding distance of the screw within the screw hole before and after loading. Screw breakage risk was evaluated by examining the maximum stress values and the frequency of stress concentrations at specific locations on the devices.

## 3 Results

### 3.1 Screw micro-movements and internal fixation device’s maximum stress values

**FIGURE 3:**
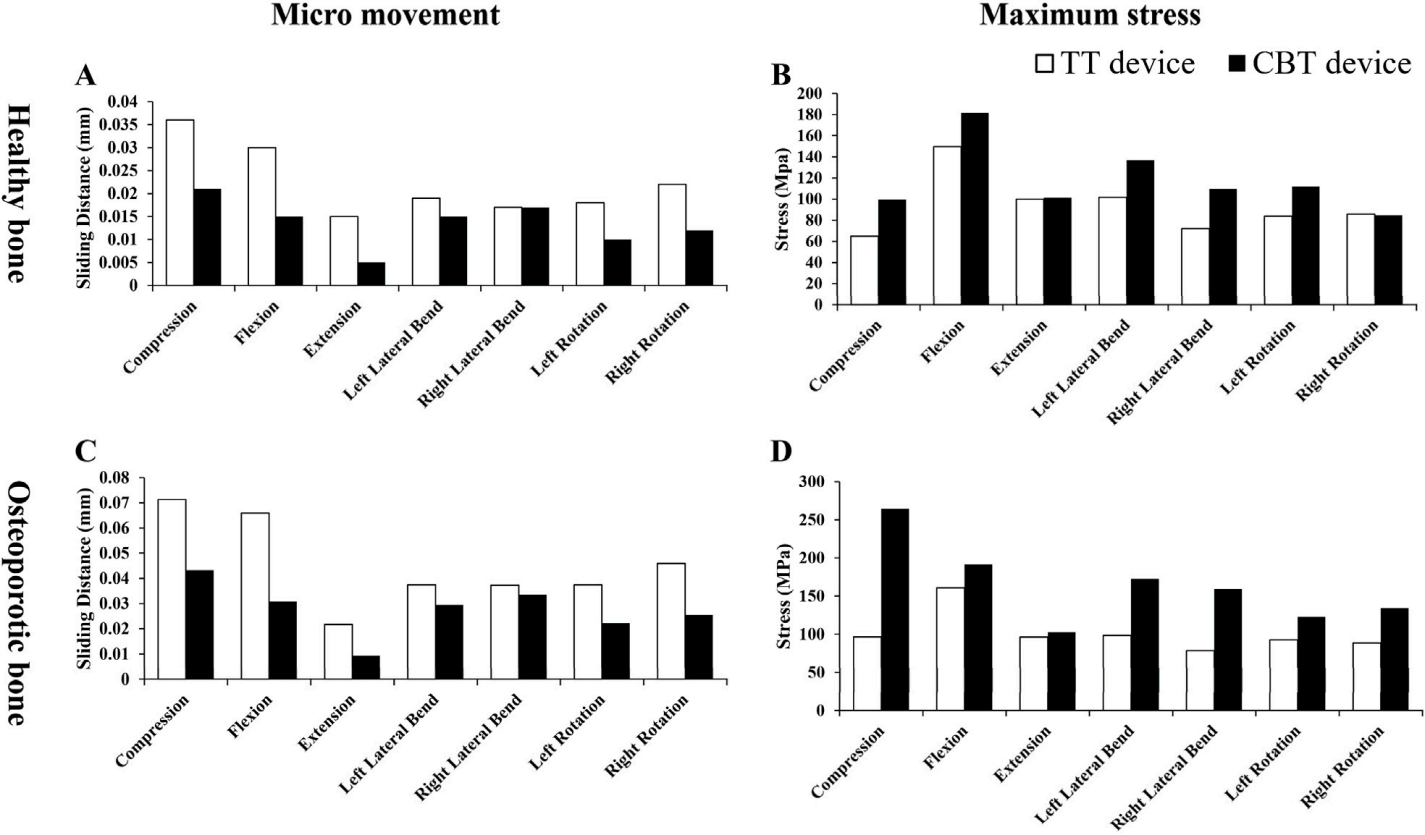
Micro-movements and maximum stress values in TT and CBT devices. Screw micro-movements in **(A)** healthy and **(C)** osteoporotic bone conditions. Maximum stress values in **(B)** healthy and **(D)** osteoporotic bone conditions.

In both healthy and osteoporotic bone, the CBT device exhibited a smaller sliding distance of the screw within the screw hole compared to the TT device across all seven motions (Figures 3A, C). Regarding maximum stress values, the CBT device experienced higher stress than the TT device in osteoporotic bone across all motions. In healthy bone, the CBT model showed lower maximum stress during right rotation compared to the TT model; however, in the other six motions, the stress on the CBT model surpassed that of the TT model (Figures 3B, D).

### 3.2 The maximum stress locations of TT and CBT device

**FIGURE 4:**
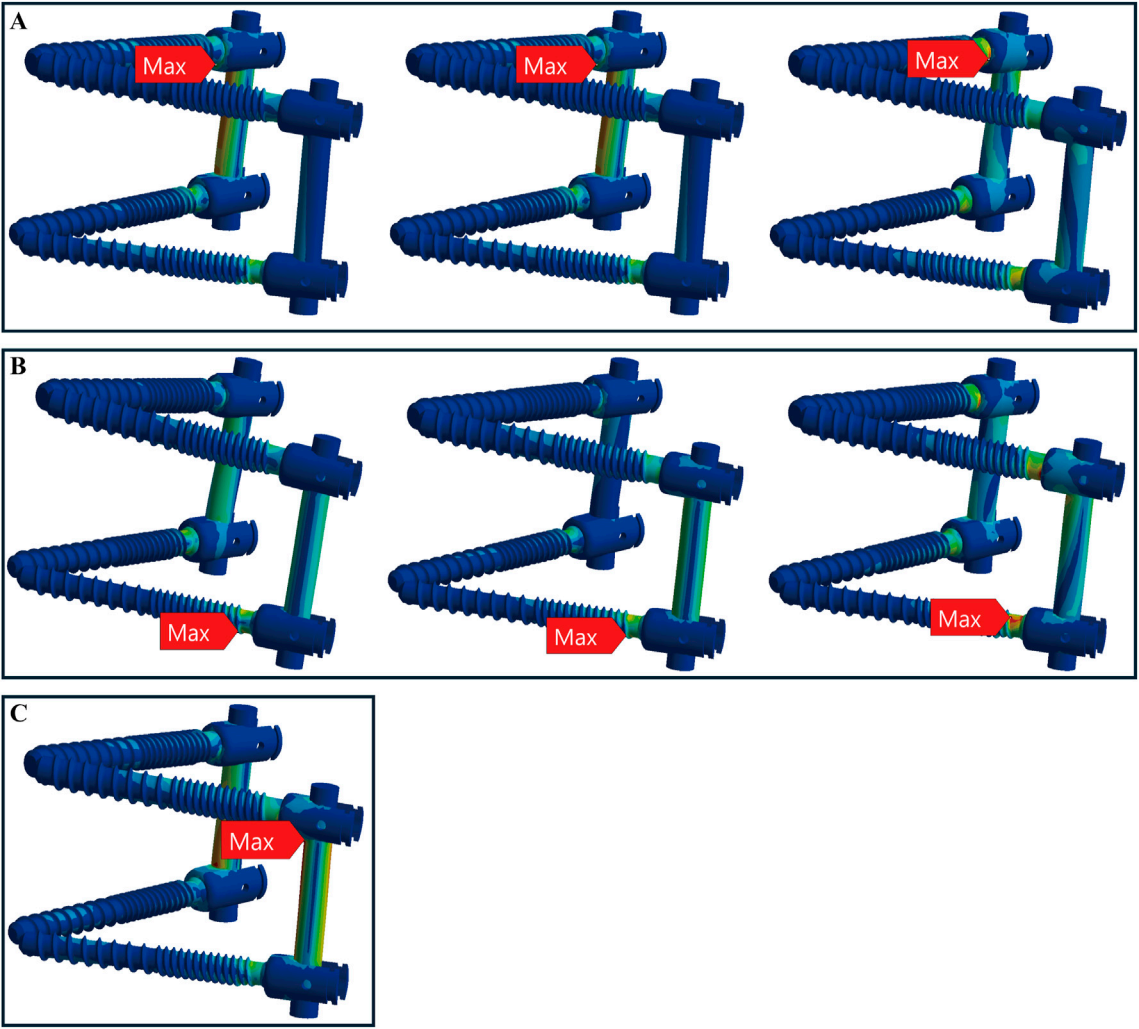
Maximum stress locations in TT device with healthy bone. Across seven motions, maximum stress occurred at three locations: **(A)** the screw head of the right L4 during flexion, right bend, and right rotation (from left to right); **(B)** the screw head of the left L5 during extension, left bend, and left rotation (from left to right); and **(C)** above the left rod during compression loading.

**FIGURE 5:**
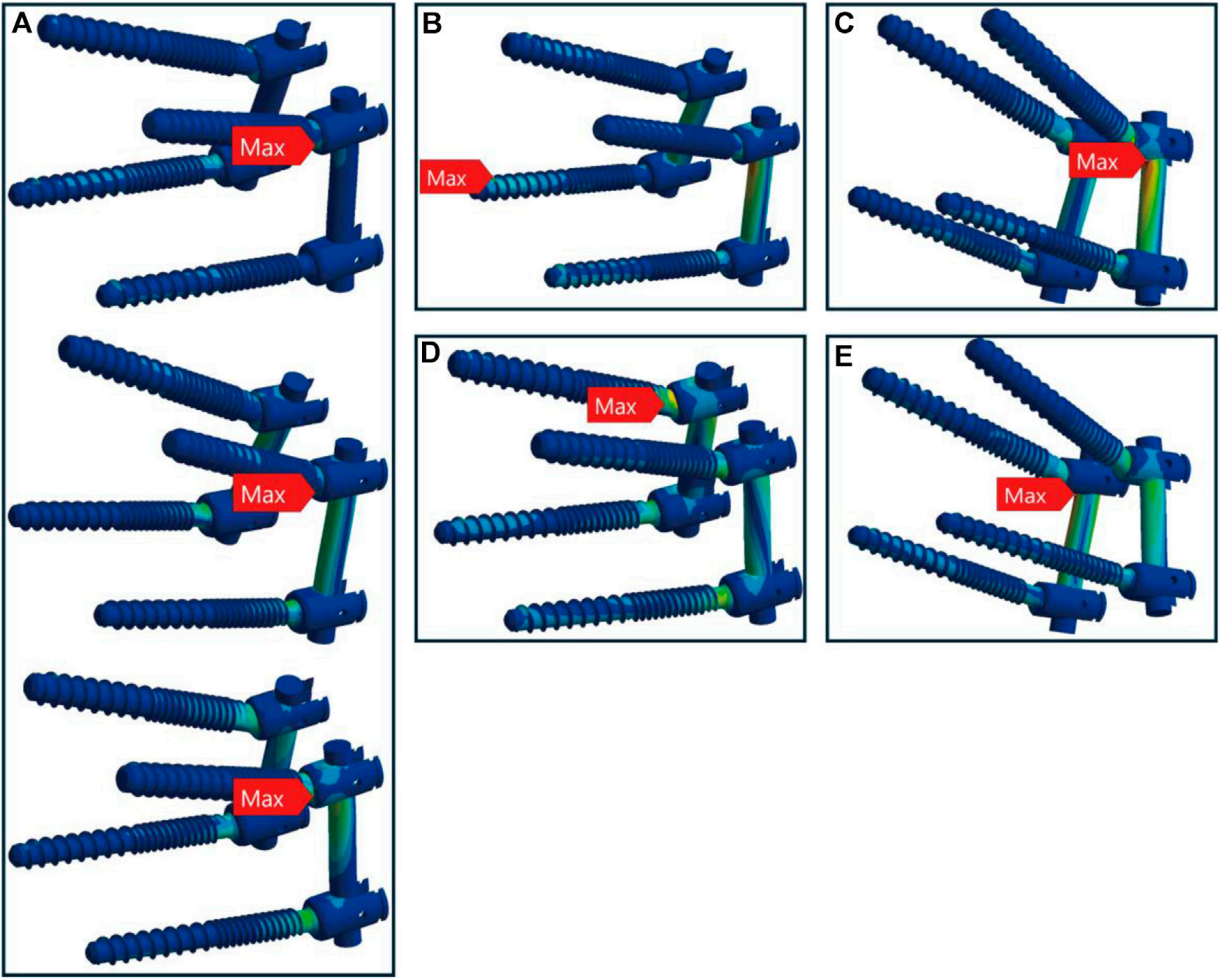
Maximum stress locations in CBT device with healthy bone. In seven motions, maximum stress occurred at five locations: **(A)** the screw head of the left L4 during flexion, extension, and left rotation (from top to bottom); **(B)** the screw tail of the right L5 during compression loading; **(C)** above the left rod during left bend; **(D)** the screw head of the right L4 during right rotation; and **(E)** above the right rod during right bend.

In healthy bone conditions, across all seven motions, the CBT device exhibited a more dispersed distribution of maximum stress locations compared to the TT device. In Figure 4, the TT device showed maximum stress at three distinct locations: the screw head of the right L4 in three motions (flexion, right bend, and right rotation), the screw head of the left L5 in three motions (extension, left bend, and left rotation), and above the left rod in one motion (pure compression). In contrast, the CBT device exhibited five different locations of maximum stress: the screw head of the left L4 in three motions (flexion, extension, and left rotation), the screw head of the right L4 in one motion (right rotation), the screw tail of the right L5 in one motion (pure compression), above the left rod in one motion (left bend), and above the right rod in one motion (right bend) (Figure 5).

**FIGURE 6:**
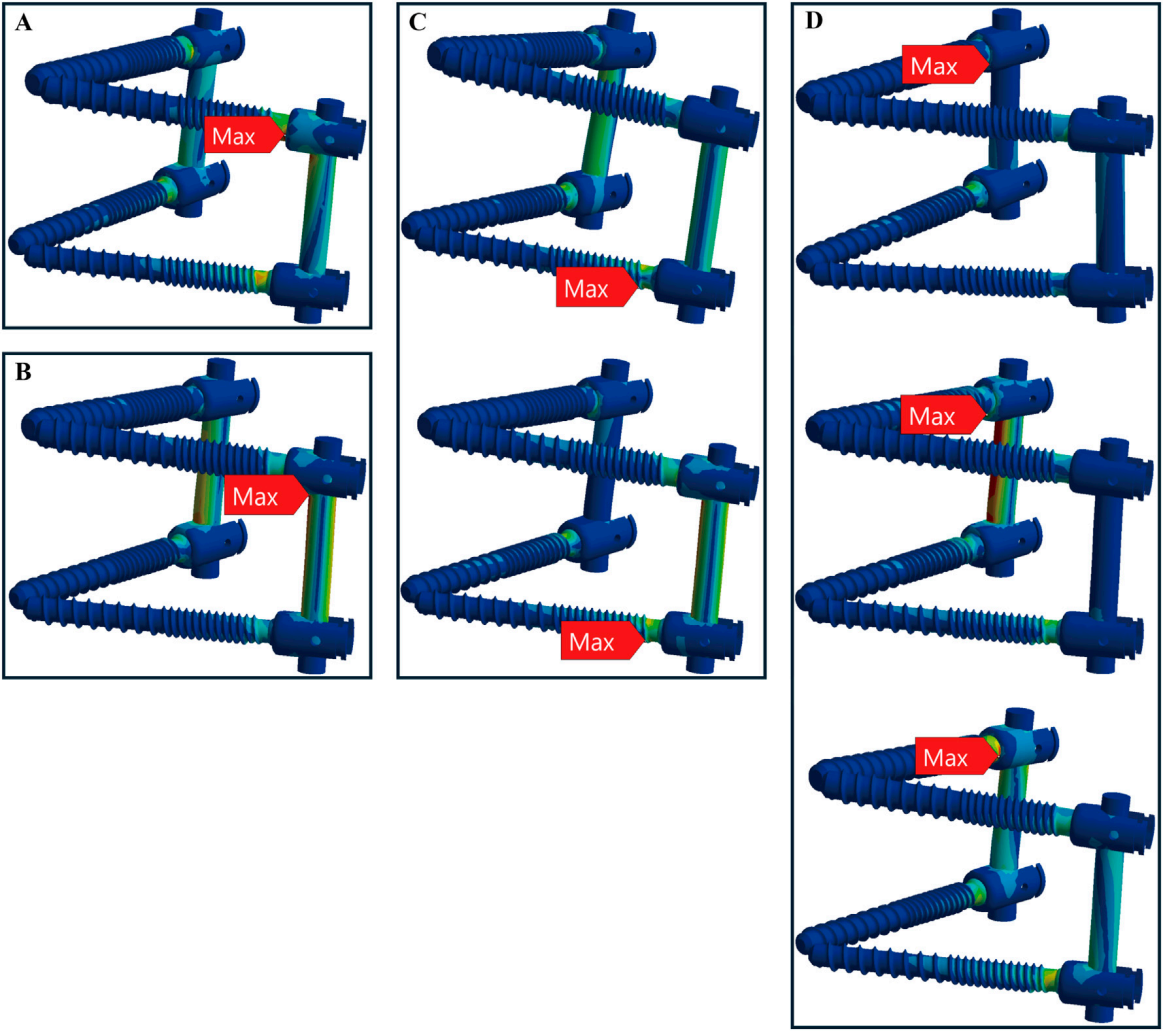
Maximum stress locations in TT device with osteoporotic bone. Across seven motions, maximum stress occurred at four locations: **(A)** the screw head of the left L4 during left rotation; **(B)** above the left rod during compression loading; **(C)** the screw head of the left L5 during extension, left bend (from top to bottom); and **(D)** the screw head of the right L4 during flexion, right bend, and right rotation (from top to bottom).

**FIGURE 7:**
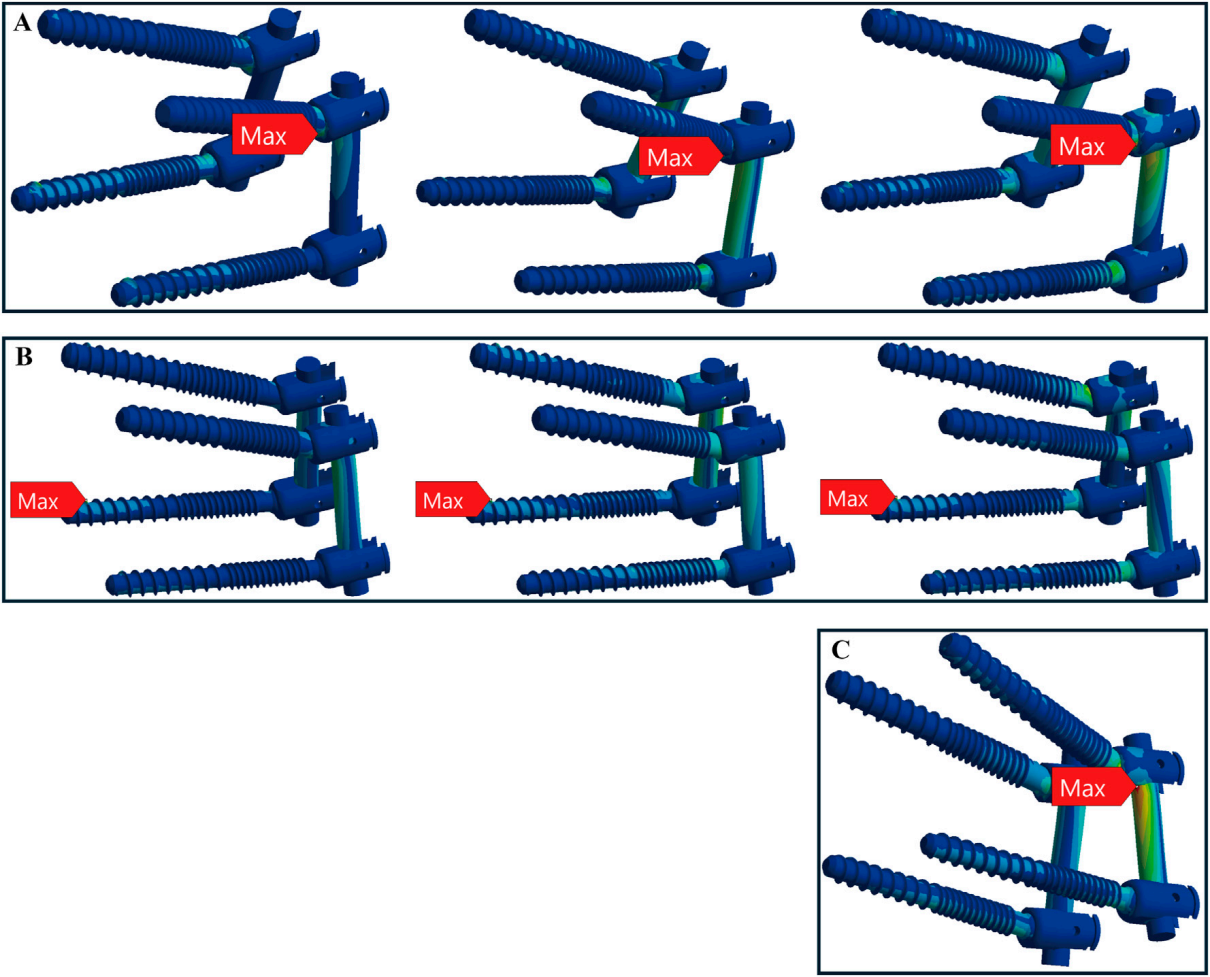
Maximum stress locations in CBT device with osteoporotic bone. In seven motions, maximum stress occurred at three locations: **(A)** the screw head of the left L4 during flexion, extension, and left rotation (from left to right); **(B)** the screw tail of the right L5 during compression, right bend, and right rotation (from left to right); and **(C)** above the left rod during left bend.

In osteoporotic bone conditions, the TT device showed maximum stress in four distinct locations across the seven motions, slightly more than the CBT device, which exhibited three locations. For the TT device, the maximum stress appeared at the screw head of the left L4 in one motion (left rotation), above the left rod in one motion (pure compression), the screw head of the left L5 in two motions (extension, left bend), and the screw head of the right L4 in three motions (flexion, right bend, and right rotation) (Figure 6). For the CBT device, the maximum stress occurred at the screw head of the left L4 in three motions (flexion, extension, and left rotation), the screw tail of the right L5 in three motions (pure compression, right bend, and right rotation), and above the left rod in one motion (left bend) (Figure 7). Notably, we find that in the TT device, six out of seven motions exhibited maximum stress at the screw head, whereas in the CBT device, three motions showed maximum stress at the screw head and three at the screw tail.

### 3.3 Model validation

Finite element models were generated using 10-node tetrahedral elements, producing meshes with 327,859 elements and 573,760 nodes. To ensure the accuracy and stability of the FE analysis results, a mesh independence test was conducted. Pure compression loads and boundary conditions from this study were applied during the testing. To confirm the convergence of the results, the maximum von Mises stress and maximum displacement were selected as key physical quantities. These quantities were compared across different mesh densities to evaluate their variation. Convergence was considered achieved when the variation in results remained below 5% across three sequential steps of mesh refinement. The final mesh size, identified through this process, was consistently applied to all models (Oefner et al., 2021).

**FIGURE 8:**
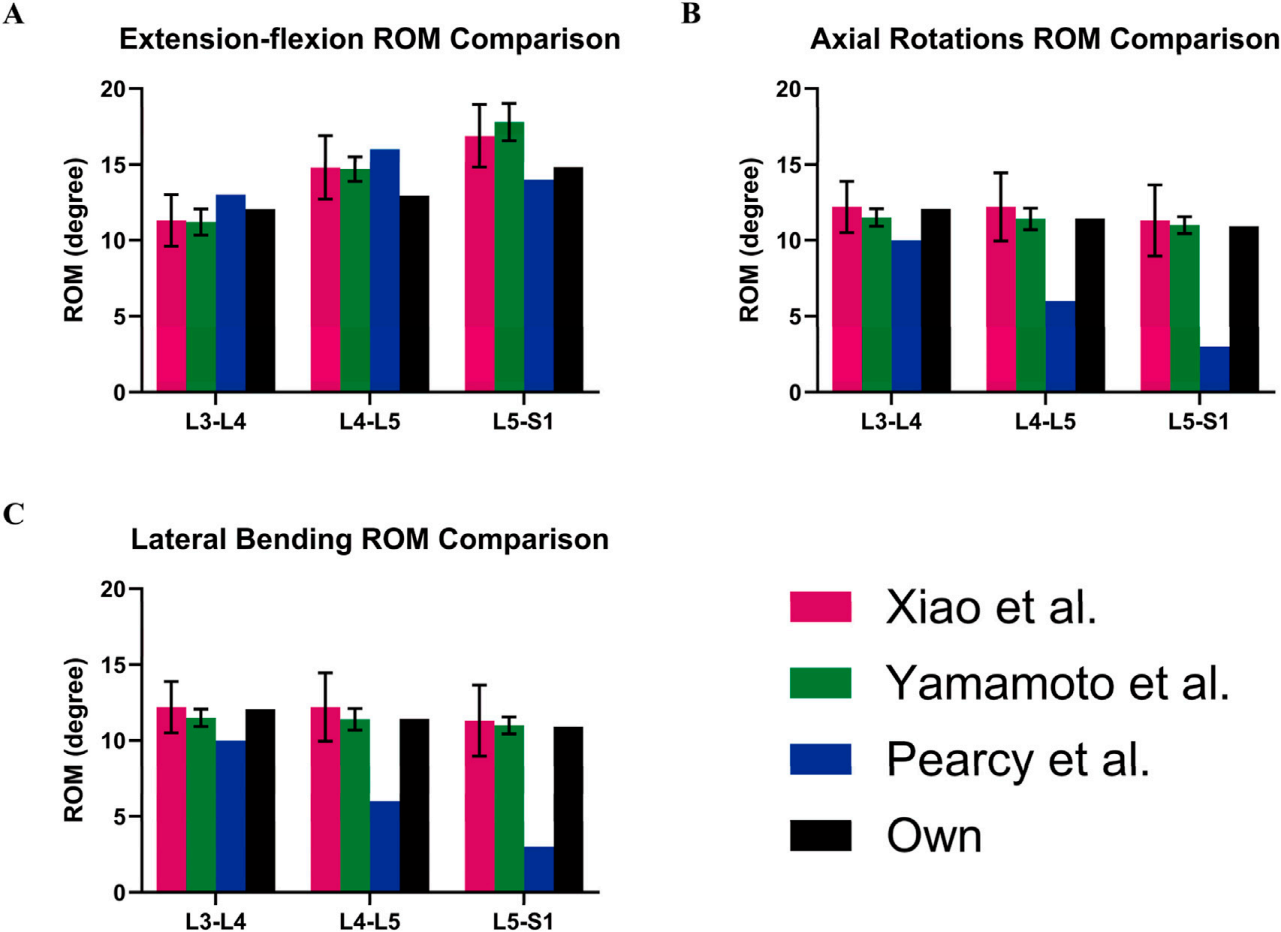
Model validation. Own model (black) compared with the ROM of spinal models from other studies: **(A)** Extension-flexion, **(B)** Axial Rotations, and **(C)** Lateral Bending.

Furthermore, the finite element analyses in this study were evaluated by calculating the average range of motion (ROM) for the L3-L4, L4-L5, and L5-S1 spinal segments. These results were then compared to previously reported data from relevant biomechanical experiments and finite element studies (Pearcy and Tibrewal, 1984; Yamamoto et al., 1989; Xiao et al., 2012). The average range of motion (ROM) for the L3-L4, L4-L5, and L5-S1 segments in our model demonstrates good consistency and reliability with previous studies. In extension-flexion motions, our results are generally consistent with those of Yamamoto et al. and Pearcy et al. In axial rotation, the results align closely with those from Yamamoto et al. For lateral bending, our model’s results are within the ranges reported by Yamamoto et al. (Figure 8). Therefore, it seems that the finite element (FE) results accurately reflect the physiological and biomechanical behavior of the spine and can be used to analyze the impact of different pedicle screw trajectories on lumbar spine stability.

## 4 Discussion

As reported in the results, we found that the CBT model exhibited smaller micro-movements of the screw within the screw hole compared to the TT model, under both healthy and osteoporotic bone conditions. This indicates that CBT screws may provide enhanced stability and a lower risk of screw loosening. Interestingly, the CBT model also displayed higher overall maximum stress values compared to the TT model, suggesting that while the CBT screws improve stability by reducing micro-movements, they are also subjected to increased stress. But this does not imply that the CBT model is more prone to breakage. In fact, our analysis revealed that the distribution of maximum stress in the CBT model is more even, particularly in healthy bone. Unlike the TT model, where maximum stress is concentrated at specific screw heads, the CBT model spreads the stress across five distinct locations, indicating a more balanced stress pattern. In osteoporotic bone, the TT model showed maximum stress at four distinct locations, while the CBT model exhibited stress concentration at three locations. The CBT model seemingly does not distribute stress as widely as the TT model. However, in the TT model, six out of seven movements concentrated maximum stress at the screw head, whereas in the CBT model, stress was evenly distributed between the screw head and screw tail across three movements each. This indicates that the screw tail in the CBT model helps to share the risk of breakage. It is well known that from a clinical standpoint. Breakage at the screw tail is less harmful than at the screw head. Therefore, even though the CBT screws experience higher stress overall, this does not necessarily imply a higher risk of failure compared to the TT screws. By distributing stress between the screw head and tail, the CBT model may potentially reduce the likelihood of implant failure.

Under healthy bone conditions, maximum stress in both the TT and CBT models was primarily concentrated at the screw head, though the CBT model displayed a more uniform distribution. In osteoporotic bone, although the TT model showed slightly more dispersed stress concentrations than the CBT model, the stress remained largely concentrated at the screw head. In contrast, the CBT model distributed nearly half of its maximum stress to the screw tail, reducing the concentration of stress at the screw head. This can also be explained using mechanical principles. When a screw is inserted into bone, the difference in material properties—such as the type of bone (healthy vs. osteoporotic)—affects the stress distribution. Additionally, if a beam has only one support, deformation and stress concentration typically occur near the support, leading to a higher concentration of stress. Similarly, in the TT model, the screw head is subject to greater stress concentration. However, when a beam has multiple supports, the compressive load is distributed more evenly across these supports, reducing stress concentration at any single point (Xu and Zhou, 2009). This is similar to the CBT model, where stress concentration around the screw is more uniform. In this case, some of the stress is transferred to the tail of the screw, which helps alleviate stress concentration at the head.

We found that the CBT model reduces screw micro-movements and prevents loosening by engaging with three layers of cortical bone, unlike the TT model, which only engages one layer. Kojima et al. supported this by demonstrating radiographically that the insertion area of CBT screws contains more cortical bone than TT screws (Kojima et al., 2015). Another study found that CBT screws rely on the posterior cortical bone for fixation, but their effectiveness is reduced in spondylolysis vertebrae due to missing cortical bone in the pedicle and lamina, confirming the importance of adequate cortical bone contact for stability (Matsukawa et al., 2016). Matsukawa’s research also identified bone density, screw length in the pedicle, and cephalad angle as key factors influencing the torque of CBT screws. His study proposed an optimal screw trajectory to maximize pedicle contact and achieve sufficient screw length within the vertebral body (Matsukawa et al., 2015a). We also adopted this optimal path to ensure maximum cortical bone contact for CBT screws. Notably, existing studies on pedicle screw loosening have often focused on pullout strength. A cadaver study showed that CBT screws had higher insertional torque and axial pullout strength than TT screws (Li et al., 2018). The recent study using lumbar vertebrae samples from Yorkshire pigs further demonstrated that screw trajectory impacts pullout strength, with the CBT enhancing screw pullout strength in this particular setting as well (Tobing and Wisnubaroto, 2020). FE-analysis allows for more comprehensive testing than *in vivo* studies. Another FE-analysis study found that CBT screws had 26.4% higher axial pullout strength than TT screws, with 27.8% and 140.2% increases in stiffness under cranial-caudal and mediolateral loading, respectively (Matsukawa et al., 2015b). This aligns with Santoni’s cadaver study, which reported a 30% increase in uniaxial pullout load for CBT screws (Santoni et al., 2009). However, in real-life situations, screws are not subjected to pure uniaxial forces. Therefore, we measured micro-movements within the screw hole during seven spinal motions to compare the loosening of CBT and TT screws. This approach better reflects actual conditions and confirms, in agreement with current studies, that CBT screws provide superior stability. Clinical studies also support these findings, showing that CBT screws perform similarly to TT screws in posterior lumbar interbody fusion and offer advantages in reducing surgical invasion and preserving nerve and muscle tissue (Gautschi et al., 2017; Marengo et al., 2018).

In summary our findings show that the CBT screws, due to their contact with more cortical bones, offer superior fixation strength and biomechanical stability but also experience higher stress loads in a FE-model of a common monosegmental L4-5 pedicle screw-intravertebral cage construct. This result aligns with several studies that report greater internal fixation stress in the CBT group compared to other fixation techniques (Zhang et al., 2021; Kahaer et al., 2022; Zhang et al., 2022). This raises a clinical concern: while CBT provides enhanced stability, could it also be more prone to fatigue fractures? Currently in cadaver fatigue test studies offer conflicting results. Under cyclical sagittal bending loads, TT screws have demonstrated superior fatigue resistance compared to CBT screws, especially in cases of poor bone quality (Akpolat et al., 2016). However, studies applying cyclical axial loads suggest that CBT screws perform better in osteoporotic spines, and they are particularly useful as a rescue technique for failed TT screw fixation (Li et al., 2018; Zhang et al., 2019). It is also important to note that existing fatigue tests primarily use single-directional loading, which does not accurately reflect real spinal movement. This study examined the frequency of maximum stress concentration during various spinal movements and found that the location of the screw tail in the apophyseal ring (Faizan et al., 2007) within the CBT model effectively redistributed the stress that would otherwise be concentrated at the screw head.

This study’s primary strength lies in its application of finite element analysis, which provided detailed biomechanical insights not easily achievable through cadaveric or clinical experiments. By simulating normal spinal movements, we obtained valuable data on screw micromotion and stress distribution, with consistent screw size across models ensuring comparability. However, in healthy bone, the CBT model showed lower maximum stress than the TT model only during right rotation, which remains unclear and requires further investigation. Additionally, this study employed homogeneous modeling, which offers computational simplicity and faster model construction, making it suitable for most preliminary analyses. However, this approach overlooks the structural differences in bone tissue, which may compromise the accuracy of the calculations. In future research, we plan to adopt heterogeneous modeling to perform more complex and detailed simulations, allowing for the inclusion of additional experimental data. Normal trunk motion relies on the complex interplay between muscle dynamics, joint mechanics, and environmental factors, enabling real-time adaptation to constantly changing surroundings (Shan et al., 2025); however, muscle and surrounding tissue dynamics were not explicitly modeled in this study. Despite these limitations, our findings offer robust evidence on the biomechanics of CBT and TT screws, contributing to a better understanding of spinal fixation stability.

Regardless, to validate the long-term effectiveness of CBT screws, dynamic analysis based on *in vivo* MRI (Wu et al., 2014), as well as larger-scale clinical and *in vitro* studies, is required. These studies should investigate key factors such as screw trajectory, insertion angle, trabecular bone morphology, and screw size, along with emerging hybrid trajectories. The long-term fatigue resistance of CBT screws under complex biomechanical loads also remains unresolved. Future research should incorporate dynamic multi-directional testing and longer patient follow-ups to determine if CBT screws offer superior stability, particularly in patients with poor bone quality. Comparative studies with TT screws and minimally invasive techniques will further clarify the potential benefits of CBT screws in treating complex spinal conditions. In addition, biomechanically, the orientation, magnitude, and direction of acting forces can significantly influence the bone’s mechanical response, including stress distribution and deformation. This study specifically examined two standard surgical paradigms, focusing on the forces generated by their distinct screw trajectories and the corresponding bone biomechanical responses. While variations in force orientation may independently impact the biomechanical response of the bone, our analysis was designed to model scenarios directly relevant to clinical practice. Future research could investigate these variations to provide a deeper understanding of their biomechanical implications.

This study underscores the potential of CBT technology to enhance spinal fixation by increasing cortical bone contact, which improves stability and reduces the risk of screw loosening and breakage. Our findings offer a new perspective and an valuable insights for advancing spinal surgery techniques. In clinical practice, combining CBT screws with technologies like intraoperative navigation or custom-made 3D guides can enhance stability and support precise screw placement, minimizing the need for extensive surgical exposure and reducing risks of muscle and nerve damage, thereby potentially improving surgical outcomes. These results of our study deepen our understanding of screw trajectory biomechanics and support the development of more reliable fixation methods, ultimately improving spinal fusion success and patient recovery.

## 5 Conclusion

The CBT model offers superior screw fixation by increasing contact with the cortical bone, reducing the risk of loosening compared to the TT model. Although CBT devices experience higher stress than TT devices, their path through three cortical bone layers—the lamina, pedicle, and apophyseal ring—offers improved integration with the vertebral body compared to the traditional pedicle screw trajectory. This, along with the screw tail’s role in mitigating the risk of breakage, results in more evenly distributed stress and improved safety from a clinical standpoint as well.

## Data Availability

The original contributions presented in the study are included in the article/supplementary material, further inquiries can be directed to the corresponding author.

## Abbreviations

TT, traditional pedicle screw trajectory; CBT, cortical bone trajectory; FE, finite element; ROM, range of motion; CT, computed tomography; ALL, anterior longitudinal ligament; PLL, posterior longitudinal ligament; LF, ligamentum flavum; SSL, supraspinous ligament; ISL, interspinous ligament.
